# Natural Orifice Translumenal Endoscopic Surgery in Humans: A Review

**DOI:** 10.1155/2012/189296

**Published:** 2012-06-06

**Authors:** Michelle P. Clark, Emad S. Qayed, David A. Kooby, Shishir K. Maithel, Field F. Willingham

**Affiliations:** ^1^Division of Digestive Diseases, Department of Medicine, School of Medicine, Emory University, Atlanta, GA 30322, USA; ^2^Department of Surgery, School of Medicine, Emory University, Atlanta, GA 30322, USA

## Abstract

Natural orifice translumenal endoscopic surgery (NOTES) had its origins in numerous small animal studies primarily examining safety and feasibility. In human trials, safety and feasibility remain at the forefront; however, additional logistic, practical, and regulatory requirements must be addressed. The purpose of this paper is to evaluate and summarize published studies to date of NOTES in humans. The literature review was performed using PUBMED and MEDLINE databases. Articles published in human populations between 2007 and 2011 were evaluated. A review of this time period resulted in 48 studies describing procedures in 916 patients. Transcolonic and transvesicular procedures were excluded. The most common procedure was cholecystectomy (682, 75%). The most common approach was transvaginal (721, 79%). 424 procedures (46%) were pure NOTES and 491 (54%) were hybrid NOTES cases. 127 (14%) were performed in the United States of America and 789 (86%) were performed internationally. Since 2007, there has been major development in NOTES in human populations. A preponderance of published NOTES procedures were performed internationally. With further development, NOTES may make less invasive surgery available to a larger human population.

## 1. Introduction

An open laparotomy is employed for many surgical procedures; however, the laparoscopic approach and minimally invasive techniques have become more common and are now preferred for certain procedures. Surgery without a cutaneous incision utilizing flexible endoscopes passed through internal organs has been termed natural orifice translumenal endoscopic surgery (NOTES). NOTES is felt to represent a logical evolution in minimally invasive surgery. NOTES is performed via a natural orifice (mouth, anus, vagina, and urethra), in some cases without requiring an abdominal wall incision. Some studies have suggested superiority over a conventional approach. NOTES had its origins in numerous small animal studies primarily examining safety and feasibility. In human trials, safety and feasibility remain at the forefront; however, additional logistic, practical, and regulatory requirements must be addressed. The purpose of this paper is to summarize and describe the progress in NOTES in humans to date.

**Historical Perspective**

Long before the term NOTES was coined, variations of the approach have been discussed in the medical literature. In 1813, the first colpotomy with a transvaginal approach to abdominal viscera was described for hysterectomy [1]. In the 1940s, gynecological procedures were performed using an endoscope passed through the recto-uterine pouch to view the pelvic organs and perform sterilization procedures [2]. Pancreatic necrosectomy was first described in 2000 and involved a controlled endoscopic perforation of the gastric wall to access the retrogastric space [3]. The concept of NOTES gained greater attention in 2004 when purposeful transgastric peritoneoscopy was performed in a porcine model [4]. The pig model was also used for tubal ligation, cholecystectomy, splenectomy, gastrojejunostomy, distal pancreatectomy, and oophorectomy with tubectomy [2, 5]. Many studies have focused on intraabdominal applications; however, intrathoracic procedures have been performed as well including mediastinoscopy, thorascopy [6, 7], and lymph node dissection [8, 9].

In 2005 a meeting occurred between members of the Society of American Gastrointestinal Endoscopic Surgeons (SAGES) and members of the American Society for Gastrointestinal Endoscopy (ASGE) to evaluate NOTES research to date and to consider challenges in NOTES development moving forward [10]. This meeting would lead to the development of the Natural Orifice Surgery Consortium for Assessment and Research [NOSCAR]. The goal of the meeting was to create a white paper setting forth concerns regarding skills and safety, research challenges, and results reporting in moving NOTES towards human populations. In 2006 the White Paper was published outlining ten critical areas that would impact the safety and appropriate usage of NOTES and the need for increased research and analysis of data. The paper identified challenges to be addressed including the physiologic implications of the procedure, safe access to the peritoneum, advancing technology and evaluating the risk of infection following NOTES [10].

**Methodology**

The review period included studies published between 2007 and 2011. These included pure and hybrid NOTES. A pure NOTES approach was defined as a procedure using flexible endoscopy without any abdominal incisions. Transvaginal NOTES was defined as a procedure where the approach involved a transvaginal conduit, often performed via a colpotomy with or without port placement. Hybrid studies were defined as surgeries utilizing flexible endoscopy combined with additional placement of one or more trocars involving flexible or rigid endoscopes [11–13]. The studies were performed in the United States of America and internationally. The transvaginal route was considered to involve incisions made near the cervix with entry into the peritoneal cavity. The transgastric route was considered to involve an endoscope passed through the mouth and esophagus and brought through a gastrotomy to enter the peritoneal cavity. Transcolonic and transvesicular approaches were not included. The literature review was completed using PUBMED and MEDLINE databases using the terms: NOTES, natural orifice translumenal endoscopic surgery, natural orifice translumenal endoscopy, human, minimally invasive surgery, NOTES in humans, and history of NOTES. Additional studies were identified in the references sections from publications located in the database search.

## 2. Results

### 2.1. Early NOTES in Humans

Pancreatic necrosectomy and pancreatic pseudocyst gastrostomy are considered by some reviewers to be the first NOTES procedures. Early reports of transgastric pancreatic procedures appeared in 2000 [3]. Recently a large multicenter retrospective study reported on the experience with pancreatic necrosectomy for walled off pancreatic necrosis [14]. In this retrospective chart review, 95 of 104 patients (91%) achieved successful resolution with a 14% complication rate. The first NOTES procedure in humans is often considered to be a transgastric appendectomy performed in India in 2006 which was presented but not reported in manuscript form [15]. This was followed by two cases of transvaginal cholecystectomy in 2006 [16] and 2007 [17]. In 2008 the first cases of transvaginal appendectomy in humans were published [18]. The results of the first pilot study for natural orifice transgastric endoscopic peritoneoscopy in humans were published in 2008 in the United States of America [19] and included ten patients with pancreatic masses who underwent diagnostic laparoscopic evaluation. These patients then underwent transgastric peritoneoscopy by surgeons blinded to the laparoscopic findings. The authors concluded that the translumenal endoscopic method is feasible, safe, and could be applied to other procedures such as appendectomy and cholecystectomy. In a more recent trial, an additional 10 patients were tested in the same manner and added to the previous cohort of 10 patients [20]. The extension of the study found a 7-minute decrease in operative time for the second cohort without significant complications related to the endoscopic approach.

### 2.2. NOTES Human Studies to Date

A compendium of published reports of NOTES in humans is presented in Table 1, grouped by procedure. Almost all these reports describe NOTES with elective indications, most commonly transvaginal cholecystectomy. Only one series describes NOTES as an emergent procedure with acute intraabdominal infection [21]. A more recent report highlights the first use of a hybrid approach for a malignant tumor of the foregut and describes a series in which the hybrid approach may have been superior to conventional approaches, beyond cosmesis and postoperative pain [22]. The literature review focused on 916 NOTES procedures published between 2007 and 2011 (Table 1). In 2007, 6 (1%) were published followed by 57 (6%) in 2008, 176 (19%) in 2009, 517 (56%) in 2010, and 160 to date (18%) in 2011. There were 721 transvaginal procedures (79%) and 195 transgastric procedures (21%). The most common procedures were cholecystectomy (682, 74%), peritoneoscopy (82,9%), and appendectomy (60,7%). Of the cholecystectomies, 612 were transvaginal (90%) and 70 were transgastric (10%). Of the peritoneoscopies, 79 were transgastric (96%) and 3 were transvaginal (4%). Of the appendectomies, 42 were transvaginal (70%) and 18 were transgastric (30%).

The most common procedures by orifice were the transvaginal cholecystectomy 4 (0.4%) in 2007, 37 (4%) in 2008, 127 (14%) in 2009, 370 (40%) in 2010, and 74 (8%) in 2011 for a total of 612 procedures (67%). This was followed by transgastric peritoneoscopy 1 (0.1%) in 2007, 20 (2%) in 2008, and 58 (6%) in 2010 for a total of 79 procedures (9%). Transgastric cholecystectomy accounted for 36 of the procedures (4%) in 2009 and 34 (4%) in 2010 for a total of 70 procedures (8%). This was followed by transvaginal appendectomy: 2 (0.2%) in 2008, 1 (0.1%) in 2009, 37 (4%) in 2010, and 2 (0.2%) in 2011 for a total of 42 (5%) of the 916 procedures. There were 424 published pure NOTES procedures (46%) and 491 hybrid NOTES procedures (54%). With regard to geography, 127 (14%) of the procedures occurred in the United States of America and 789 (86%) internationally.

Overall complication rates varied by procedure type and access site. The complication rate was 0% for the following procedures: transvaginal peritoneoscopy, transvaginal appendectomy, transgastric and transvaginal gastrectomy, transvaginal nephrectomy, transvaginal colectomy, transgastric gastric mass resection, transgastric stapled cystogastrostomy, transvaginal splenectomy, transvaginal incisional hernia repair, transgastric PEG rescue, and transvaginal liver and ovarian biopsy and may reflect the small sample size reported to date. The complication rate for transvaginal cholecystectomy ranged from 1.5% to 25% while that for transgastric peritoneoscopy was 12.5%. The rate for transgastric cholecystectomy was 18% and in both transgastric appendectomy and gastric banding was 33.3%.

### 2.3. International Multicenter Trial on Clinical Natural Orifice Surgery

The international multicenter trial on clinical natural orifice surgery or NOTES IMTN study analyzed data on NOTES procedures from July 2007 to June 20, 2009 [23]. A total of 362 NOTES patients were followed. The study was conducted in 16 centers in 9 countries including Brazil, Peru, Ecuador, Chile, Italy, Germany, Mexico, India, and Cuba. General surgeons performed most of the procedures. The most common procedures were transvaginal cholecystectomy (66%) and transvaginal appendectomy (10%). Four of the centers performed transgastric procedures, accounting for 12% of the total. The overall complication rate was 8.8% (6.9% for transvaginal and 23.2% for transgastric procedures). All 43 procedures involving the transgastric approach were hybrid procedures. There were no mortalities.

### 2.4. German National Registry

The German Registry for NOTES is a privately funded registry that was started in March of 2008 [24]. It collects data voluntarily and directly from surgeons performing NOTES at their respective facilities. Data collected include patient demographics, target organs, therapy, and postoperative outcome. The results of the first 14 months of the registry were published [24]. The operations were documented between March 2008 and April 2009. General surgeons performed 97% of the procedures with a small number utilizing a gynecologist. Of the 551 patients, 534 used rigid endoscopes and 99% were hybrid procedures and all were transvaginal. As in the IMTN Study, cholecystectomy was the most common, accounting for 85% of the procedures. The complication rate was 3% and conversions to open or laparoscopic surgery occurred in 5%. There was no reported mortality. Advanced patient age and obesity were associated with increased conversion rates but were not associated with an increase in complication rates. The authors also concluded that transvaginal hybrid NOTES cholecystectomy is a practicable and safe alternative to laparoscopic cholecystectomy [24].

### 2.5. Patient Acceptance

There were 3 studies reviewed regarding patient opinions about NOTES. In a study published in 2009, a survey about NOTES and laparoscopic surgery was distributed to 192 presurgical patients [25]. They rated the importance of different potential benefits of NOTES versus laparoscopic surgery for cholecystectomy. It was found that risk of postoperative complication, recovery time, and postoperative pain was more important to patients than cost, visual scar, length of hospital stay, or anesthesia type (*P* < 0.001). When the patients were asked which method of surgery they preferred, 56% reported NOTES and 44% reported laparoscopic surgery. Patients felt they could have less pain, cost, risk of complication, and recovery time than with open or laparoscopic surgery. They also felt that more skill and training were required for NOTES than for other surgical methods (*P* < 0.04). Patients who had completed some college preferred NOTES. Patients who were 70 years of age and older, as well as patients who had previously undergone flexible endoscopy preferred laparoscopic surgery to NOTES (*P* < 0.04). In a study published in 2008, a hundred patients with an intact gallbladder who were undergoing EUS or ERCP for evaluation for abdominal complaints were asked about their preference between a laparoscopic or a NOTES cholecystectomy [26]. The patients were given a questionnaire about laparoscopic cholecystectomy and were then given a detailed description of the NOTES procedures using oral, rectal, and vaginal conduits. 78% of patients preferred NOTES over the traditional laparoscopic approach. Patients with age less than or equal to 50 years (odds ratio [OR] 1.3, *P* = .61), female sex (OR 2.1, *P* = .14), and prior endoscopy experience (OR 2.2, *P* = .19) preferred NOTES to laparoscopic surgery. As was seen previously when the laparoscopic approach was compared to open surgery, patients similarly may prefer NOTES to laparoscopy provided that the complication rates were comparable. The oral orifice appeared to be the preferred conduit [26]. In a study that reported on transvaginal NOTES procedures in a group of 100 women, 87% preferred transumbilical laparoendoscopic single-site surgery, while 8% preferred laparoscopy and only 4% preferred a transvaginal approach. Reasons cited included postoperative fear of complications with fertility and sexuality. Postoperative abstinence from intercourse following a transvaginal NOTES procedure was a concern in 76% of women who believed this could make them feel less feminine, less attractive, and could cause tension with their partners [27].

## 3. Discussion

NOTES is evolving as a feasible and acceptable alternative to more traditional surgical approaches, and the experience continues to grow. In this paper, published reports of NOTES in humans increased from 6 in 2007 to 517 in 2010. Despite studies suggesting that patients prefer an oral route [26], the transvaginal approach is by far the most common NOTES approach (79%) for both pure and hybrid procedures. The gallbladder remains the most common target organ in pure and hybrid NOTES (75%). Patients appear to prefer NOTES to laparoscopic surgery provided that a similar complication rate is achieved. Hybrid NOTES is common in humans, comprising 54% of reported cases. Human NOTES procedures were reported internationally in 27 countries. The preponderance of NOTES procedures were performed internationally with 86% of reported NOTES cases abroad and 14% in the United States.

### 3.1. The Transvaginal Approach

A transvaginal approach has been the most frequently utilized despite a number of challenges. This is in likelihood due to the ease and ready availability of a standard closure method for the transvisceral incision, frequently the colpotomy. In this paper the transvaginal approach was utilized in 79% of reports and was the most frequent approach for both pure and hybrid NOTES procedures. Gynecologists have been performing colpotomies for many years, providing ample experience with this surgical technique and the subsequent closure. Nevertheless, patients do not tend to prefer the transvaginal approach. In one study reviewed here, only 4% preferred a transvaginal approach when compared to single site or a laparoscopy. Patients express concern for decreased fertility and sexuality. Additionally, a transvaginal approach is only possible in half the population. As NOTES continues to evolve, enabling technologies may make closure of alternative visceral incisions more feasible.

### 3.2. Hybrid NOTES

Hybrid NOTES is common in humans, comprising 54% of reported cases in this paper. A hybrid approach is felt to be safer given the presence of standard transabdominal instruments to address potential complications. Hybrid approaches also enable standard-of-care closures of visceral incisions, leak testing, and additional visibility. Furthermore, the combination of laparoscopic and endoscopic techniques may enable more novel surgeries and may allow movement beyond cholecystectomy. Hybrid procedure and NOTES may have the potential to move beyond the recapitulation of standard and safe surgeries such as cholecystectomy, enabling more novel techniques with greater potential benefits over the traditional approach [28, 29].

### 3.3. Complications

Multiple potential benefits have been suggested for NOTES procedures including decreased postoperative wound infection, faster recovery, less intraabdominal adhesions, less postoperative ileus, decreased incidence of incisional hernias, less postoperative pain, and better cosmesis. Surgical wound infections are not an uncommon complication after traditional open or laparoscopic surgeries, occurring in up to 20% of patients undergoing intraabdominal surgery [30]. NOTES could also prove useful when transabdominal routes are not optimal or are difficult, such as in morbidly obese patients, patients with abdominal wall infections, or in the critically ill patients with contraindications to general anesthesia [28]. Many of the studies reviewed here reported no complications [9, 18, 21–23, 31–44]. The most common reported complications were sepsis, hematoma, laceration, perforation, and biliary leakage (Table 1). For the most common procedure, transvaginal cholecystectomy, the complication rate ranged from 1.5–25%. The main limitation presently is the lack of comparative data from trials comparing one approach with another in a prospective manner [45, 46].

### 3.4. NOTES Technology

Technology remains a challenge; much of the equipment and device technology used to date has been repurposed from other applications. Equipment typically employed in NOTES was not designed for use intraperitoneally [11]. The tools are not designed to manipulate the intraabdominal organs and they often have insufficient angulation and push force via small accessory channels [47]. There are also questions about safety, particularly with the gastric closure, for management of complications and regarding compression syndromes [10]. Endoscope design, conduit access, assist devices, and systems for closure require reengineering and redesign for optimal function in the NOTES setting [46]. This requires industry activity, investment, and interest. Following an initial flurry of interest, active development by industry has fluctuated but remains a critical component to progress.

### 3.5. Regulations

Multiple regulatory requirements will contribute to the penetrance of NOTES into the general human population. Transitioning to human studies requires IRB oversight and justification in utilizing a NOTES approach over a traditional standard. The risk of a novel procedure must be justified against a presumed potential benefit with a new approach. Similarly, device development is associated with rigorous regulatory requirements. A substantial contribution to the technology needed for NOTES procedures comes from small startup companies [48]. Devices of the past were often approved with the FDA 510 K pathway, and physicians have used devices in nontraditional ways. This system is changing and newer devices are going through the longer, more expensive premarket approval application (PMA) process. Following the PMA process, a procedure or device must pass through the current procedural terminology (CPT) coding pathway, third-party-payer process, and hospital and purchasing requirements [48]. Presently, NOSCAR is encouraging dialogue between the multiple parties. If NOTES continues to show that it is a safe, minimally invasive procedure with faster recovery times and more patient acceptance it may be advantageous to payers and third parties to work towards wider acceptance [48].

### 3.6. Training

There is considerable debate about who should be trained to perform NOTES among general surgeons, thoracic surgeons, gynecologists, and gastroenterologists. In this paper, the majority of human NOTES procedures were performed by general surgeons. Regardless of the specialty, the operator should have expertise with intra-and extralumenal anatomy, flexible endoscopy, and/or laparoscopy, and undergo specialized training to learn the techniques. As techniques move in and out of the operating room, in and out of the endoscopy suite, and away from or towards the patient's bedside, it becomes less certain which specialist should perform or train in which procedure [29].

## 4. Conclusion

Natural orifice translumenal endoscopic surgery has progressed to human populations and is evolving for certain indications. Due to practical concerns, much of the initial work has focused on elective procedures. Many NOTES surgeries have redemonstrated laparoscopic procedures which have a high degree of safety and little morbidity. More recent studies have raised the possibility that NOTES may come to offer more substantial improvements over the current standard, going beyond cosmesis and reduced pain medication usage [22]. The studies reviewed here suggest a high degree of safety and feasibility with low rates of infection. As the field progresses, rigorous, prospective, controlled studies will become more important in defining the exact benefits versus a traditional approach [73]. With greater experience in redemonstrating standard procedures, it is hoped that the field will continue to evolve, enabling novel approaches that distinguish the potential for more unique contributions.

## Figures and Tables

**Table 1:** Published NOTES studies in human populations between 2007 and 2011, grouped by procedure.

Procedure	Year	Route	*N* (range and total)	Complication rate (Range)
Cholecystectomy [16, 17, 21, 23, 49–66]	2007–2011	TV	1–240 Total = 612	1.5%–25% (Abscess, hematuria, subhepatic collection, sepsis, hematoma, laceration, perforation, biliary leakage)

Cholecystectomy [23, 62, 67–69]	2009-2010	TG	4–29 Total = 70	18% (sepsis, hematoma, laceration, perforation, biliary leakage)

Peritoneoscopy [19, 20, 31, 45, 70]	2007, 2008, 2010, 2011	TG	1–40 Total = 79	12.5% (infection, bleeding, wound dehiscence)

Peritoneoscopy [21, 32]	2008, 2011	TV	1-2 Total = 3	0%

Appendectomy [18, 34]	2008–2011	TV	1–37 Total = 42	0%

Appendectomy [23, 60, 71]	2009-2010	TG	1–14 Total = 18	33.3% (pneumothorax)

Gastrectomy (partial) [9, 23, 33, 37, 42]	2011	TG	14	0%

Gastrectomy [23, 33, 37, 42]	2008–2010	TV	Sleeve = 1–5 Partial = 2 Total = 12	0%

Nephrectomy [23, 40, 41]	2009-2010	TV	1–5 Total = 10	0%

Colectomy [35, 36, 38]	2008–2010	TV	1–12 Total = 16	0%

Gastric mass resection [22]	2011	TG	7	0% No recurrence to date

Gastric banding [72]	2010	TV	3	33.3% (ureter damage)

Cancer staging [23]	2010	TV	8	

Stapled cystogastrostomy [44]	2011	TG	6	0%

Gynecologic surgery [23]	2010	TV	11	

Splenectomy [39]	2009	TV	1	0%

Incisional hernia repair [43]	2010	TV	1	0%

Hepatic cystectomy [23]	2010	TV	1	

PEG rescue [31]	2007	TG	1	0%

Liver, ovary biopsy [32]	2008	TV	1	0%

TV: transvaginal, TG: transgastric.
